# Prognostic factors for local recurrence in patients with rectal cancer submitted to neoadjuvant chemoradiotherapy and total mesorectal excision

**DOI:** 10.1016/j.clinsp.2024.100464

**Published:** 2024-08-09

**Authors:** Caio Sergio Rizkallah Nahas, Sergio Carlos Nahas, Carlos Frederico Sparapan Marques, Ulysses Ribeiro Junior, Leonardo Bustamante-Lopez, Guilherme Cutait Cotti, Antonio Rocco Imperiale, Rodrigo Ambar Pinto, Ivan Cecconello

**Affiliations:** a Universidade de São Paulo (USP), São Paulo, SP, Brazil; b AdventHealth Orlando, Florida, United States

**Keywords:** Rectal cancer, Chemoradiotherapy, Neoadjuvant therapy, Total mesorectal excision, Prognosis, Survival

## Abstract

Prognostic factors for local recurrence in patients with rectal cancer submitted to neoadjuvant chemoradiotherapy and total mesorectal excision.

**Background:**

The standard curative treatment for locally advanced rectal cancer of the middle and lower thirds is long-course chemoradiotherapy followed by total mesorectal excision.

**Purpose:**

To evaluate the prognostic factors associated with local recurrence in patients with rectal cancer submitted to neoadjuvant chemoradiotherapy and total mesorectal excision.

**Methods:**

Retrospective study including patients with rectal cancer T3–4N0M0 or T (any)*N* + M0 located within 10 cm from the anal border, or patients with T2N0M0 located within 5 cm, treated by long course chemoradiotherapy followed by total mesorectal excision with curative intent. Clinical, demographic, radiologic, surgical, and anatomopathological data were collected. Local recurrence was estimated using the Kaplan-Meier function, and risk was estimated according to each characteristic using univariate and multivariate analyses.

**Results:**

270 patients were included, 57.8% male and mean age 61.7 (30‒88) years. At initial staging, 6.7% of patients were stage I, 21.5% stage II, and 71.8% stage III. Open surgery was performed in 65.2%, with sphincter preservation in 78.1%. Mortality within 30 postoperative days was 0.7%. After 49.4 (0.5‒86.1) months of median follow-up, overall and local recurrences were 26.3% and 5.9%. On multivariate analyses, local recurrence was associated with involvement of the mesorectal fascia on restaging MRI (HR = 9.11, *p* = 0.001) and with pathologic involvement of radial surgical margin (HR = 8.19, *p* < 0.001).

**Conclusion:**

Local recurrence of rectal cancer treated with long-course chemoradiation and total mesorectal excision is low and is associated with pathologic involvement of the radial surgical margin and can be predicted on restaging MRI.

## Highlights

• Rectal cancer recurrence occurs predominantly in distant organs.
• Local recurrence of rectal cancer could be predicted on restaging MRI.
• Rectal cancer recurrence of patients treated with long course chemoradiation and total mesorectal excision is low and is associated with pathologic involvement of the radial surgical margin.

## Background

Since the introduction of a multidisciplinary approach for the treatment of primary locally advanced middle and lower rectal cancer based on improvement in radiological imaging, Chemoradiation Therapy (CRT), and Total Mesorectal Excision (TME), a significant improvement has been accomplished on oncologic outcomes.^1^^,^^2^ However, 2.4%‒10% of patients will develop local failure.^3^, ^4^, ^5^, ^6^, ^7^

Rectal cancer prognoses can be particularly affected by other factors beyond AJCC TNM stage,^8^^,^^9^ such as tumor response to neoadjuvant treatment, involvement of Circumferential Resection Margin (CRM), involvement of the intersphincteric plane, presence of Extramural Venous Invasion (EMVI), lateral pelvic lymph nodes, and presence of mucinous component.^10^, ^11^, ^12^ In particular, a positive CRM is associated with increased risk for Local Recurrence (LR) (5-year local recurrence: HR = 3.50; *p* < 0.05;^12^, ^13^, ^14^ CRM involvement increases the likelihood of LR after (TME) by more than 4-times.^15^ Therefore, TME should typically be performed as a part of a low anterior resection or Abdominoperineal Resection (APR). A 2-cm distal mural margin is usually adequate for distal rectal cancers when combined with TME. For tumors located at or below the mesorectal margin, a 1 cm distal mural margin can be acceptable only for those who have achieved a good response to CRT.^16^

Despite all these concerns, high-volume centers still present some local failures even after specialized multidisciplinary care. The aim of this study is to identify the prognostic factors associated with local recurrence in patients with rectal cancer submitted to neoadjuvant chemoradiotherapy and total mesorectal excision in a single institution.

## Methods

This retrospective study was approved by the Institutional Board Review of the studied institution (9,076,078). It included consecutive patients with a biopsy-proven rectal adenocarcinoma located within 10 cm from the anal verge, stage T3–4N0M0 or T(any)*N* + M0, and patients with T2N0 of the distal rectum (0‒5 cm from the anal verge, because of the risk of needing an APR) from a prospectively collected database of the studied institution where a randomized trial was set to assess non-inferiority of the non-operative management compared to immediate surgical resection with TME in patients with Clinical Complete Response (cCR) after neoadjuvant CRT (NCT02052921)^17^ from October 2011 to November 2015. Clinical complete response was defined as an absence of tumor simultaneously on three exams: digital rectal examination, colonoscopy and high-resolution MRI. Non-operative management was offered only for cCR patients. Nearly complete responders were not eligible for non-operative management. Patients with synchronous colorectal cancer or other non-colorectal cancers, rectal cancer in the setting of inflammatory bowel disease or familial adenomatous polyposis, significant delay (> 8 weeks after restaging) on performing surgery, palliative resections and patients with previous chemotherapy or radiotherapy were excluded. Patients with cCR randomized for nonoperative management were also excluded.

Patients with cCR randomized for operative management were operated in the same manner as patients who did not achieve cCR.

**Fig. 1:**
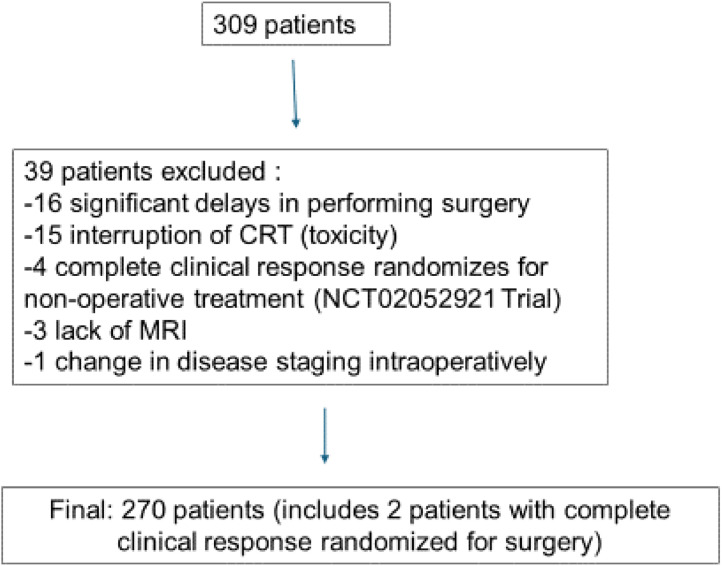
Flowchart.

A total of 309 consecutive patients with primary rectal adenocarcinoma without metastatic disease were treated at the studied institution. Data from 39 patients were excluded for the following reasons: 16 for significant delays in performing surgery, 15 for interruption of CRT related to toxicity, four for being selected for non-operative treatment according to another study at the institution (NCT02052921),^17^ three due to lack of preoperative MRI, and one case due to the palliative nature of the surgery due to a change in disease staging intraoperatively (Fig. 1).

Patients were staged and re-staged by the same colorectal surgeon and clinical oncologist by digital exam, colonoscopy, pelvic high-definition MRI, thorax and abdominal CT scans. The total dose of pelvic radiation was 5040 Gy given in 30 sessions (180 Gy/day, only on weekdays, along 5.6 weeks) All patients received 5-FU based chemotherapy with leucovorin by IV bolus on days 1 to 5, concomitant with radiation in weeks 1 and 5. *Re*-stage occurred 8 weeks after completion of CRT. Staging and restaging high-resolution MRI and CT scans were evaluated by only two radiologists who were specifically dedicated to this task. The radiologist who has seen the staging exam also reviewed the restaging exam not blinded to the pretreatment stage.

The MRI Tumor Response Grade (TRG) was adopted as described elsewhere.^3^ Clinical suspicion of lateral pelvic sidewall node was primarily based on lymph node size (short axis > 0.7 cm for internal iliac or obturatory node on staging MRI, short axis > 0.6 and > 0.4 respectively for obturator and internal iliac nodes on restaging MRI).

All patients who did not achieve a cCR were submitted to TME. Surgeries were performed two weeks after restaging. All surgical procedures were performed at the studied institution by one of four surgeons who had previous training in rectal cancer surgery. All patients underwent curative or potentially curative TME, with high ligation of the inferior mesenteric artery. Resection of adjacent organs was performed en bloc with the rectum when firm adhesions or macroscopic tumor invasion were present. The decision to perform an APR was based on oncological factors (risk of involvement of distal margin), and/or sphincter invasion. All surgical specimens were staged according to the American Joint Committee on Cancer guidelines. After surgery, all patients were assessed by digital exam, proctoscopy, and CEA level, every 3 months in the first three years, and every 6 months in the 4th and 5th years thereafter. Pelvic high-definition MRI, and CT scans of thorax and abdomen were performed every 4 months in the first year, and then every 6 months. A colonoscopy was performed once a year.

The following data were collected:

a) Gender, age, body mass index.
b) Staging MRI characteristics: T stage (mri T2 vs. mri T3/4), N stage (mriN- vs. mriN+), MF, mucinous component, EMVI, intersphincteric plane, lateral pelvic lymph nodes, and TRG (good response TRG 1‒3 vs. poor response TRG 4, 5).
c) Surgical data: surgical access (open versus laparoscopic), sphincter preservation, en bloc resection of adjacent organs.
d) Surgical specimen characteristics: T-stage, N stage, lymphovascular invasion, perineural invasion, pathologic response (complete vs. incomplete), lymph node yield (< 12 vs. ≥ 12), involvement of distal and/or circumferential margin, mesorectum integrity (complete/nearly complete vs. incomplete).

By reviewing the electronic medical record, it was assessed whether there was local or systemic tumor recurrence, with a specific description of the affected site or organ. The starting point for the analyses of survival and recurrence was the day of surgery. The diagnosis of recurrence was confirmed according to the presence of radiological findings and/or clinical evolution and/or elevation of tumor markers, or through histological confirmation (biopsy or surgical resection). LR was defined as any type of recurrence (tumor, lymph node, peritoneal implant) at any pelvic site, whether in the anastomosis, perineum, perirectal tissues, genitourinary tract (bladder, prostate, seminal vesicle, ureter, vaginal wall), pre-sacral region, sacrum, coccyx, lateral pelvic walls, obturator or internal iliac lateral pelvic lymph nodes.

### Statistical analysis

The qualitative characteristics evaluated in all patients were described using absolute and relative frequencies.

The LR was evaluated using the Kaplan-Meier function and compared the time according to the characteristics of interest using log-rank tests. The risks of recurrence were estimated according to each characteristic evaluated using a bivariate Cox regression with the respective 95% Confidence Intervals Multiple Cox regression was performed including the variables that had a lower descriptive level than 0.10 (*p* < 0.10) in the bivariate tests, keeping in the final model only the jointly significant variables, using the stepwise backward method with entry and exit criteria at 5%.

The software IBM-SPSS for Windows version 20.0 was used to carry out the analyzes and the software Microsoft Excel 2003 was used to tabulate the data. The tests were performed with a significance level of 5%.

## Results

Of the 270 patients included in the study, 156 (57.8%) were male and the mean age was 61.7 (30‒88) years. As for initial staging, 18 (6.7%) patients were stage I (T2N0), 58 (21.5%) were stage II, and 194 (71.8%) were stage III. Most surgeries were performed via open access 176 (65.2%), sphincter was preserved in 211 (78.1%) cases, and en bloc resection of adjacent organs was necessary in 34 (12.6%) patients (eight cases of partial resection of the vagina, seven of the uterus, seven of the seminal vesicles, five of the uterus combined with the vagina, four of the prostate associated with the bladder, and three of the uterus associated with the ureter).

The involvement of surgical margins in the anatomopathological evaluation of the specimens occurred in 14 (5.2%) cases (13 cases radial, one case of radial and distal involvement; therefore, were all included as “radial margin involvement” in the statistical analysis). The average number of LN yield was 22.9.

Lateral pelvic lymph node dissection was performed in 18 cases and positive involvement was confirmed in the anatomopathological evaluation in 5 of them.

Detailed clinical and radiological characteristics are presented in Table 1, while the characteristics of the surgical procedures and the anatomopathological evaluation of the surgical specimens are described in Table 2.

**Table 1:** Clinical and radiological characteristics of 270 patients.

Variable	n (%)
**Gender**	
Male	156 (57.8)
Female	114 (42.2)
**Age**	
< 50 years old	39 (14.4)
≥ 50 years old	231 (85.6)
**BMI (Kg/m^2^)**	
< 30	227 (84.1)
≥ 30	43 (15.9)
**mriT stage**	
T2	52 (19.3)
T3, T4	218 (80.7)
**mriN stage**	
N0	76 (28.1)
*N*+	194 (71.9)
**mri Mucinous component**	
No	205 (75.9)
Yes	65 (24.1)
**mri EMVI**	
No	113 (41.9)
Yes	157 (58.1)
**mri FM**	
Negative	165 (61.1)
Positive	105 (38.9)
**mri intersphincteric plane**	
No	244 (90.4)
Yes	26 (9.6)
**mri pelvic sidewall lymph node involvement**	
No	224 (83)
Yes	46 (17)
**ymriT stage**	
T0, 1, 2	101 (37.4)
T 3, 4	169 (62.6)
**ymriN stage**	
N0	173 (64.1)
*N*+	97 (35.9)
**TRG**	
TRG 1, 2, 3	143 (53)
TRG 4, 5	127 (47)
**ymri Mucinous component**	
No	223 (82.6)
Yes	47 (17.4)
**ymri EMVI**	
No	150 (55.6)
Yes	120 (44.4)
**ymri FM**	
Clear	181 (67)
Involved	89 (33)
**ymri intersphincteric plane**	
Clear	249 (92.2)
Involved	21 (7.8)
**ymri pelvic sidewall lymph node involvement**	
No	247 (91.5)
Yes	23 (8.5)

BMI, Body Mass Index.

**Table 2:** Characteristics of surgical procedures and anatomopathological evaluation of surgical specimens of the 270 patients.

Variable	n (%)
**Surgical access**	
Open	176 (65.2)
Laparoscopic	94 (34.8)
**Sphincter preservation**	
No	59 (21.9)
Yes	211 (78.1)
**Multivisceral en bloc resection**	
No	236 (87.4)
Yes	34 (12.6)
**ypT stage**	
T 0, 1, 2	137 (50.7)
T 3, 4	133 (49.3)
**ypN stage**	
N0	186 (68.9)
*N*+	84 (31.1)
**Lymphatic invasion**	
No	234 (86.7)
Yes	36 (13.3)
**Perineural invasion**	
No	217 (80.4)
Yes	53 (19.6)
**Pathologic complete response**	
No	46 (17)
Yes	224 (83)
**Lymph node yield**	
≥ 12	233 (86.3)
< 12	37 (13.7)
**Radial Margin**	
Clear	256 (94.8)
Involved	14 (5.2)
**Mesorectum integrity**	
Complete/nearly complete	248 (91.8)
Incomplete	22 (8.2)

Data in number (n) and percentage (%).

After a median follow-up time of 49.4 (0.5‒86.1) months, there were 65 (24.1%) deaths, 35 (13%) due to the disease and 30 (11.1%) due to other causes.

A total of 71 (26.3%) patients had general recurrence (local and/or systemic), 16 (5.9%) cases of LR, and 59 (21.9%) cases of systemic relapse (four patients had LR and systemic).

LR occurred in the anterior pelvic compartment in five cases (four in the vagina and one in the prostate/bladder), in the anastomotic or perianastomotic region in four cases, in the lateral compartments in four cases (internal iliac lymph node chain) and in the posterior region (pre sacral) in three cases.

On univariate analyses, stage ymriT3/4, ymriEMVI+, ymriMF+, non-preservation of the sphincter, presence of perineural invasion and involvement radial margin (*p* < 0.05) were associated with local recurrence (Tables 3 and 4). However, on multivariate analyses, only ymriMF+ and involvement of the radial margin in the surgical specimen were associated with local recurrence (Table 5).

**Table 3:** Univariate analysis for local recurrence according to demographic and radiological characteristics.

Variable	Estimated medium time (months)	95% CI		HR	95% CI		Local recurrence	N total	%	p
		Inferior	Superior		Inferior	Superior				
**Gender**										0.328
Male	82.7	80.2	85.2	1.00			7	156	4.5	
Female	80.2	76.7	83.6	1.63	0.61	4.38	9	114	7.9	
**Age**										0.297
< 50 years old	78.7	71.8	85.7	1.00			4	39	10.3	
≥ 50 years old	81.9	79.8	84.0	0.55	0.18	1.71	12	231	5.2	
**BMI (Kg/m²)**										0.800
< 30	81.9	79.6	84.1	1.00			13	227	5.7	
≥ 30	73.6	68.8	78.3	1.18	0.34	4.13	3	43	7.0	
**mriT Stage**										**0.033**
T2	80.9	80.9	80.9	1.00			0	52	0.0	
T3, T4	80.6	78.0	83.2	29.67	0.19	4693.83	16	218	7.3	
**mriN Stage**										0.462
N0	77.6	74.6	80.5	1.00			3	76	3.9	
*N*+	81.3	78.7	83.8	1.60	0.45	5.60	13	194	6.7	
**mri Mucinous component**										0.422
No	81.7	79.4	84.0	1.00			11	205	5.4	
Yes	80.4	75.6	85.1	1.54	0.53	4.42	5	65	7.7	
**mri EMVI**										**0.011**
No	84.5	83.0	86.1	1.00			2	113	1.8	
Yes	79.4	76.0	82.7	5.54	1.26	24.37	14	157	8.9	
**mri MF**										**<0.001**
Clear	85.0	83.6	86.3	1.00			3	165	1.8	
Involved	75.6	70.8	80.4	7.85	2.24	27.57	13	105	12.4	
**mri Intersphincteric plane**										
Clear	82.2	80.1	84.3	1.00			13	244	5.3	
Involved	77.8	69.4	86.1	2.20	0.63	7.72	3	26	11.5	
**mri pelvic sidewall lymph node involvement**										
No	82.1	80.1	84.2	1.00			11	224	4.9	
Yes	78.2	71.5	84.8	2.27	0.79	6.54	5	46	10.9	
**ymriT Stage**										**0.005**
T0, 1, 2	82.5	81.4	83.7	1.00			1	101	1.0	
T3, 4	79.3	76.0	82.6	10.42	1.38	78.89	15	169	8.9	
**ymriN Stage**										0.318
N0	75.6	73.0	78.1	1.00			12	173	6.9	
*N*+	83.1	80.1	86.0	0.57	0.18	1.76	4	97	4.1	
**TRG**										0.714
1, 2, 3 (good response)	76.6	74.2	79.1	1.00			8	143	5.6	
4, 5 (poor response)	81.4	78.2	84.6	1.20	0.45	3.21	8	127	6.3	
**ymri Mucinous component**										0.319
No	82.1	79.9	84.3	1.00			12	223	5.4	
Yes	78.7	72.4	85.1	1.77	0.57	5.48	4	47	8.5	
**ymri EMVI**										**0.006**
No	81.5	79.9	83.0	1.00			4	150	2.7	
Yes	78.3	74.1	82.5	4.25	1.37	13.18	12	120	10.0	
**ymri MF**										**<0.001**
Clear	85.0	83.7	86.3	1.00			3	181	1.7	
Involved	74.6	69.1	80.1	9.88	2.82	34.68	13	89	14.6	
**ymri Intersphincteric plane**										
Clear	81.7	79.6	83.9	1.00			15	249	6.0	
Involved	71.8	65.5	78.1	0.84	0.11	6.35	1	21	4.8	
**ymri pelvic sidewall lymph node involvement**										
No	82.0	79.8	84.1	1.00			14	247	5.7	
Yes	72.0	65.1	78.8	1.67	0.38	7.36	2	23	8.7	

Log-rank test; bivariate Cox regression.

BMI, Body Mass Index; FM, Mesorectal Fascia; EMVI, Extramural Venous Invasion; TRG, Tumor Regression Grade.

**Table 4:** Univariate analysis for local recurrence according to surgical and specimen characteristics.

Variable	Estimated medium time (months)	95% CI		HR	95% CI		Local recurrence	N total	%	p
		Inferior	Superior		Inferior	Superior				
**Surgical access**										0.174
Open	80.7	77.8	83.5	1.00			13	176	7.4	
Laparoscopic	79.0	76.8	81.2	0.43	0.12	1.51	3	94	3.2	
**Sphincter preservation**										**0.006**
No	76.1	70.0	82.2	1.00			8	59	13.6	
Yes	83.3	81.3	85.2	0.27	0.10	0.73	8	211	3.8	
**Multivisceral en bloc resection**										0.228
No	82.2	80.2	84.3	1.00			13	236	5.5	
Yes	77.5	68.8	86.2	2.13	0.61	7.49	3	34	8.8	
**ypT stage**										0.079
yp T 0, 1, 2	80.8	78.8	82.8	1.00			5	137	3.6	
yp T 3, 4	79.8	76.2	83.4	2.50	0.87	7.19	11	133	8.3	
**ypN stage**										0.219
ypN0	82.2	80.0	84.4	1.00			9	186	4.8	
ypN+	79.7	75.2	84.3	1.84	0.69	4.95	7	84	8.3	
**Limphatic invasion**										0.069
No	82.0	80.0	84.0	1.00			12	234	5.1	
Yes	77.1	68.8	85.3	2.75	0.88	8.55	4	36	11.1	
**Perineural invasion**										**0.038**
No	82.8	80.7	84.8	1.00			10	217	4.6	
Yes	76.9	70.4	83.4	2.79	1.01	7.68	6	53	11.3	
**complete pathologic response**										0.564
No	81.5	79.2	83.8	1.00			14	224	6.3	
Yes	78.2	74.4	82.0	0.65	0.15	2.85	2	46	4.3	
**Lymph node yield**										0.944
< 12	74.5	69.1	79.9	1.00			2	37	5.4	
≥ 12	81.8	79.6	84.0	0.95	0.22	4.18	14	233	6.0	
**Surgical Radial margin**										**<0.001**
Free	82.9	81.1	84.8	1.00			11	256	4.3	
Compromised	53.8	40.6	67.0	9.54	3.31	27.54	5	14	35.7	
**Mesorectum integrity**										
Complete/nearly complete	83.1	81.2	84.4	1.00			14	248	5.6	0.183
Incomplete	79.4	69.7	83.2	2.21	0.83	5.48	2	22	9.1	

Log-rank test; bivariate Cox regression.

**Table 5:** Multivariate analysis for local recurrence.

Variable	HR	95% CI		p
		Inferior	Superior	
ymri Mesorectal Fascia+ (involved)	9.11	2.59	32.10	0.001
Surgical Radial margin (involved)	8.19	2.78	24.10	< 0.001

Cox multiple regression stepwise backward method.

**Fig. 2:**
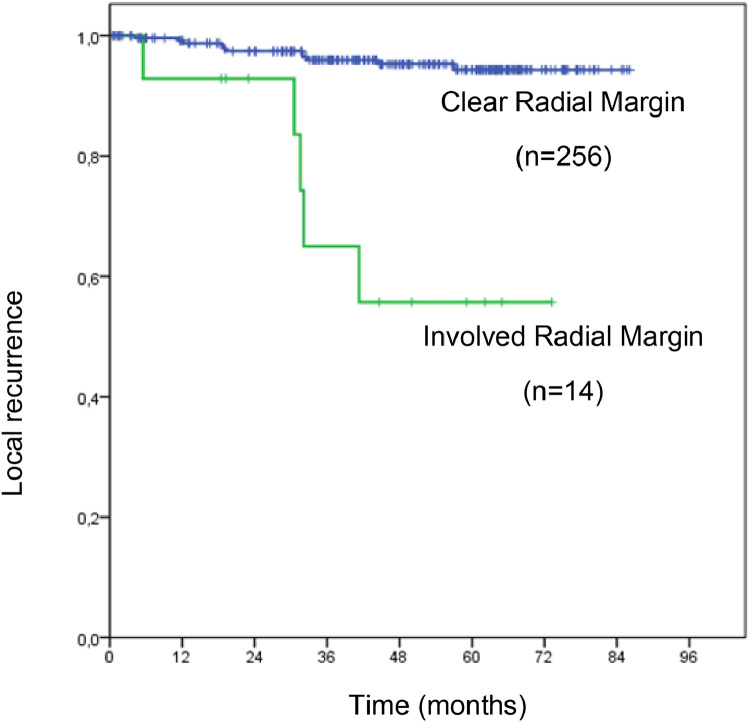
Estimated Kaplan-Meier function of local recurrence probability according to surgical radial margin involvement.

Fig. 2 shows the estimated Kaplan-Meier function of local recurrence probability according to surgical radial margin involvement.

The 59 patients with systemic recurrence occurred in the following sites: exclusively pulmonary in 31 cases, exclusively hepatic in 16, exclusively cerebral in two, exclusively peritoneal in two, exclusively mediastinal in one, and combined (more than one site) in seven cases (hepatic + lung in four cases, bone + lung in one case, lung + brain in one case, and liver + brain in one case). In total, the most affected sites were the lungs (37 cases/62.7%) and liver (21 cases/35.6%). The mean overall DFS estimate was 65.8 months (95% CI 61.9–69.8 months).

Surgical-related mortality occurred in 4 (1.5%) patients: two cases due to colorectal anastomosis leakage (despite protective ileostomy) evolving with secondary complications and death on the 27th PO day and 36th PO day, respectively; one case of acute myocardial infarction on the 1st PO day, progressing to renal failure and death after 63 days of hospitalization; and one case of urinary anastomosis fistula associated with abdominal wound complications in a patient submitted to TME and en bloc prostatectomy that evolved to death on the 44th PO day.

## Discussion

This study demonstrated that the oncological results obtained at the studied institution were adequate, with emphasis on the low LR rate (5.9%) compatible with what is reported in the literature (2.4%‒10%).^3^, ^4^, ^5^, ^6^, ^7^ The relapse of patients in this study was predominantly systemic (21.9%), with the lungs (62.7%) and liver (35.6%) being the main sites affected, in line with the literature data.^18^, ^19^, ^20^, ^21^

As for local recurrences, it is worth remembering that 4 of the 16 cases (25%) occurred in the internal iliac lymph nodes, which cannot be attributed to inappropriate TME. Lateral pelvic lymphadenectomy is still a controversial subject, but it has been gaining strength in recent years. While its routine performance is not recommended in the US, some Japanese centers have been performing it prophylactically to treat patients with lower rectal cancer without the use of radiotherapy.^22^ Although the feasibility of lateral lymphadenectomy after CRT in Western patients with higher BMI has been demonstrated recently, there are some concerns about its morbidity and doubts about its benefits.^23^

As for the staging of the patients in this series, it is noteworthy that most patients were advanced on admission (71.9% stage III), which still reflects the difficulty of early diagnosis and/or difficulty in obtaining a quick referral and specialized care for patients with rectal cancer in the Unified Health System of the studied country.

Regarding the evaluation of MRI characteristics in the staging and restaging of patients, it is noteworthy that the mriT and mriN stages were not associated with a worse prognosis for patients in the current study, in contrast to the five-year survival estimates according to the AJCC TNM classification.^1^ A possible explanation could be the large disproportion in the number of patients with stages rmT3/4 (80.7%) and rmN+ (71.9%) in this sample.

As for the importance of evaluating MF by MRI, the MERCURY study clearly demonstrated worse 5-year survival in the group of patients with compromised MF (42.2% vs. 62.2%) and was the only prognostic factor proven to be associated with worse OS, DFS and LR in the multivariate analysis.^24^^,^^25^ In the present study, the authors also observed MF involvement as a predictor of LR.

Regarding the presence of EMVI on MRI, although the authors did not find a direct independent association with a worse prognosis in the studied sample, it has been identified as a relevant factor in the literature. In a study by Yu et al.,^26^ 78% of patients who had evidence of EMVI on their staging MRI were significantly less likely to respond to neoadjuvant CRT (HR = 2.5). However, neoadjuvant CRT was able to change the EMVI status from positive to negative after CRT was shown to be associated with better 3-year DFS (88%) when compared to those who remained positive (46%, *p* < 0.0001). Smith et al.^27^ demonstrated that the 3-year DFS was 35% for mriEMVI-positive patients compared to 74.1% for mriEMVI-negative patients.

Another important study by the MERCURY Group[5] demonstrated that involvement of the intersphincteric plane assessed by MRI, as well as positive CRM, extramural invasion greater than 5 mm, and the presence of EMVI were recognized as the worst risk factors for LR. It suggests that patients with stage T3 without these conditions regardless of the suspicion of lymph node disease in the MRI evaluation, can be surgically treated without neoadjuvant therapy. In the present study, all patients, even those without these risk factors, were treated with neoadjuvant CRT. However, in this series involvement of the intersphincteric plane both on MRI before and after CRT was not associated with a worse prognosis for recurrence.

Sphincter preservation was possible in 78% of these patients, although it was not an independent prognostic factor. In the literature, variable results regarding recurrence rates for APR have been reported. Data from the National Surgical Adjuvant Breast and Bowel Project (NSABP) R-01 revealed a 5% LR rate for patients undergoing an APR.^28^ A retrospective analysis of 14 studies identified positive CRM in 10% of APR samples compared to 5% of LAR samples.^29^ Among these patients, LR rates were high (20% vs. 11%) and 5-year survival was worse (59% vs. 70%) in patients with APR compared to those with LAR. However, it is assumed that the tumors of patients undergoing APR tend to be lower and more locally advanced at the time of the operation. Over the past decade, the improvement of extrasphincteric APR has led to a reduction in the involvement of radial surgical margin and LR. A Dutch study with wider APR resections proved equivalent data of CRM involvement when compared to LAR in 190 patients.^30^

Although it was necessary in only 12% of the cases, the combined en-bloc resection of other organs also did not confer a worse prognosis on the patients, despite presuming that these were cases of larger and more invasive tumors. However, it is known from the literature that the major oncological determinant of LR is obtaining an R0 resection,^31^^,^^32^ which was possible in 82.3% of these cases of multivisceral en-bloc resection.

The present study has some limitations. The first is its retrospective nature, with no review of MRI imaging reports or anatomopathological evaluation of surgical specimens. Another aspect previously discussed was the non-uniformity in the indication of lateral pelvic lymphadenectomy, although the oncological benefits of this procedure are still under debate in the literature. Finally, the authors know that several tissue or serum molecular biomarkers, such as research on circulating tumor cells, research on specific gene expression profiles, expression of proteins such as the epidermal growth factor receptor and vascular endothelial growth factor, research on mutations and DNA mutilations, among others, have been widely studied in terms of their ability to predict prognosis and response to chemoradiotherapy treatment, although they were not considered in the current study due to the limited availability in this service.

## Conclusions

Local recurrence of rectal cancer treated with long-course chemoradiation and total mesorectal excision is low and is associated with pathologic involvement of the radial surgical margin and can be predicted on restaging MRI.

## Conflicts of interest

The authors declare no conflicts of interest.
